# Guidance strategies for a participatory ergonomic intervention to increase the use of ergonomic measures of workers in construction companies: a study design of a randomised trial

**DOI:** 10.1186/1471-2474-15-132

**Published:** 2014-04-17

**Authors:** Steven Visser, Henk F van der Molen, Judith K Sluiter, Monique HW Frings-Dresen

**Affiliations:** 1 Coronel Institute of Occupational Health, Academic Medical Center, University of Amsterdam, P.O. Box 22660, Amsterdam 1100 DD, The Netherlands; 2 Arbouw, P.O. Box 213, Harderwijk 3840 AE, The Netherlands

**Keywords:** Participatory ergonomics, Ergonomic measures, Physical work demands, Construction industry

## Abstract

**Background:**

More than seven out of 10 Dutch construction workers describe their work as physically demanding. Ergonomic measures can be used to reduce these physically demanding work tasks. To increase the use of ergonomic measures, employers and workers have to get used to other working methods and to maintaining them. To facilitate this behavioural change, participatory ergonomics (PE) interventions could be useful. For this study a protocol of a PE intervention is adapted in such a way that the intervention can be performed by an ergonomics consultant through face-to-face contacts or email contacts. The objective of this study is to evaluate the effectiveness of the face-to-face guidance strategy and the e-guidance strategy on the primary outcome measure: use of ergonomic measures by individual construction workers, and on the secondary outcome measures: the work ability, physical functioning and limitations due to physical problems of individual workers.

**Methods/Design:**

The present study is a randomised intervention trial of six months in 12 companies to establish the effects of a PE intervention guided by four face-to-face contacts (N = 6) or guided by 13 email contacts (N = 6) on the primary and secondary outcome measures at baseline and after six months. Construction companies are randomly assigned to one of the guidance strategies with the help of a computer generated randomisation table. In addition, a process evaluation for both strategies will be performed to determine reach, dose delivered, dose received, precision, competence, satisfaction and behavioural change to find possible barriers and facilitators for both strategies. A cost-benefit analysis will be performed to establish the financial consequences of both strategies. The present study is in accordance with the CONSORT statement.

**Discussion:**

The outcome of this study will help to 1) evaluate the effect of both guidance strategies, and 2) find barriers to and facilitators of both guidance strategies. When these strategies are effective, implementation within occupational health services can take place to guide construction companies (and others) with the implementation of ergonomic measures.

**Trail registration:**

Trailnumber: ISRCTN73075751, Date of registration: 30 July 2013.

## Background

Of all Dutch construction workers (i.e., the blue collar workers), 74% report their work as being physically demanding [1]. The use of ergonomic measures has proven to reduce exposure towards physical risk factors, such as trunk flexion, manual handling, and kneeling e.g. [2-4] as proxy for decrease in musculoskeletal disorders. However, few construction workers use ergonomic measures in daily practice e.g., [5,6].

To reduce the exposure towards physical risk factors, different strategies are used, such as informational, educational, and facilitative strategies [7]. These strategies or combinations of strategies influence the process of reducing physical risk factors. This process consists of different behavioural phases; awareness of the risk factors; attitude towards ergonomic measures; change of behaviour; and the ability to change the behaviour. Construction workers must pass through these behavioural phases before they can actually make use of the ergonomic measures. In addition to the construction workers, other actor groups within the construction company must also pass the behavioural phases to facilitate the use of ergonomic measures [7,8].

One way to facilitate behavioural changes is through participatory ergonomic (PE) interventions e.g., [7,9,10]. The basic concept of the PE interventions is to involve workers in adapting their workplaces to reduce injury and increase productivity [11]. With the involvement or participation of workers, it is hoped that difficulties encountered in changing working methods by means of the behavioural phases may be overcome. Although differences in effectiveness of PE interventions on the use of ergonomic measures are found [12,13], a review on the effectiveness of PE interventions in improving health outcomes – such as musculoskeletal symptoms, injuries, and reduction in lost days from work or sickness absence – found partial to moderate evidence that PE interventions had a positive impact [14]. However, a large randomised controlled trial did not confirm this finding for musculoskeletal disorders [15]. In a review of Van Eerd *et al*. [16] it was found that 83% of the 52 included studies implemented other tools and equipment and that 85% found a positive effect of the intervention on reducing physical risk factors, increasing productivity/output and positive health outcomes.

Within the review of van Eerd *et al*. [16], fifteen out of 16 studies found that the presence of a PE specialist, defined as an ergonomics consultant, was a facilitator to the PE intervention process. The role of this ergonomics consultant was firstly to initiate and then to guide the PE intervention process [16]. In this study, we want to investigate the effect of two types of guidance strategies on the use of ergonomic measures, as well as on health outcomes. In addition, we want to evaluate the process and the costs-benefits of both guidance strategies. The two guidance strategies are: 1) a face-to-face guidance strategy in which the ergonomics consultant guides the PE intervention through face-to-face contacts with the PE team; and 2) an e-guidance strategy in which the ergonomics consultant guides the PE intervention by means of e-mail contacts with the PE team.

We hypothesise that the face-to-face guidance strategy will increase the use of ergonomic measures, work ability, and physical functioning while decreasing limitations due to physical problems more when compared to the e-guidance strategy. This would be due to a higher compliance to the face-to-face guidance strategy compared with the e-guidance strategy, as a result of the presence of the ergonomics consultant within the face-to-face guidance strategy. Although the financial costs will be higher in the face-to-face guidance strategy, due to more time spent by the ergonomics consultant during the guidance sessions, more financial benefits will be gained through reduced physical risk factors due to an increase in the use of ergonomic measures.

### Objectives

The objective of this study is to assess the effectiveness of two guidance strategies for a PE intervention: a face-to-face guidance strategy and an e-guidance strategy. The effectiveness will be assessed on 1) the primary outcome measure concerning the use of ergonomic measures by individual construction workers, and the secondary outcome measures concerning health outcomes: work ability, physical functioning and limitations due to physical problems of individual construction workers; 2) a process evaluation, and 3) a cost-benefit evaluation.

## Methods/Design

For the description of the design of the participatory ergonomic intervention and the two guidance strategies, we follow the CONSORT statement [17,18].

### Study design

A randomised intervention trial with a follow-up at six months is performed to compare the effectiveness of the two ergonomic guidance strategies, a face-to-face guidance strategy and an e-guidance strategy, of a PE intervention. The effectiveness of both guidance strategies will be compared on the use of ergonomic measures, work ability, physical functioning, limitations due to physical problems of individual construction workers, a process evaluation and a cost-benefit evaluation.

According to the researchers, the study protocol did not met the criteria of the “Act medical-scientific research with human participants” and therefore, the Medical Ethics Committee of the Academic Medical Center Amsterdam (AMC) was not asked for approval of the study protocol.

### Setting

The guidance strategies will be given to construction companies. Inclusion criteria of the construction companies are: 1) less than 50 employees (small and medium enterprises within the Dutch construction industry); 2) performing physically demanding work; and 3) having the potential to improve the use of ergonomic measures among their workers to reduce work demands/risks for work-related musculoskeletal disorders.

### Study population

The research population includes all construction workers (i.e. blue collar workers) within the participating companies.

### Recruitment of participants

Four methods of recruiting the construction companies will be used. First of all, the occupational health services approach, i.e. Employers of construction workers for whom risk factors for work-related health complaints are known will be approached to participate in this study. Dutch construction workers have the opportunity to visit occupational physicians for periodic health examinations. Arbouw (the Health and Safety Institute for the Dutch construction industry) keeps records of construction workers for whom risk factors for work-related health complaints have been detected [19]. Construction companies will receive a letter from the researchers containing a description of the study containing a globally description of the intervention, the costs for the companies, and what is expected of the companies during the intervention. One week after sending the letter, the companies will be contacted for their decision. In addition, occupational physicians and ergonomics consultants working for Occupational Health Services will be asked to approach construction companies within their customer base. Construction companies nominated by the occupational physicians and ergonomics consultants will then be contacted by the researchers.

The second method of recruitment is the Labour Inspectorate approach and consists of sending a letter to construction companies within the inclusion criteria who received a warning or a penalty in 2012 from the Dutch Labour Inspectorate with a description of the study containing a globally description of the intervention, the costs for the companies, and what is expected of the companies during the intervention. Construction companies will then contact the researchers for participation in this study.

The third method of recruitment is to contact the National Board of Employers of four physically demanding occupations (masonry, concrete reinforcement work, plastering, and floor layers). Stakeholders within these national boards will be informed about the study and asked to recruit companies to participate in this study. Construction companies recruited by the national boards will be contacted by the researchers.

The final method of recruitment is to contact construction companies within the personal network of the researchers or who have previously performed work for the Academic Medical Center, Amsterdam.

When construction companies are willing to participate, a meeting between the researcher (SV) and the companies CEO is established. During this meeting the construction companies will be informed about the purpose, the measurements, the two types of intervention and the randomized allocation to one of the intervention groups. Furthermore, they are informed that the costs of the ergonomics consultants will not be charged to the companies during the intervention. The only costs for the companies are the costs for renting or buying ergonomic measures. Construction companies agree to participate by signing an informed consent.

### Interventions

**Figure 1:**
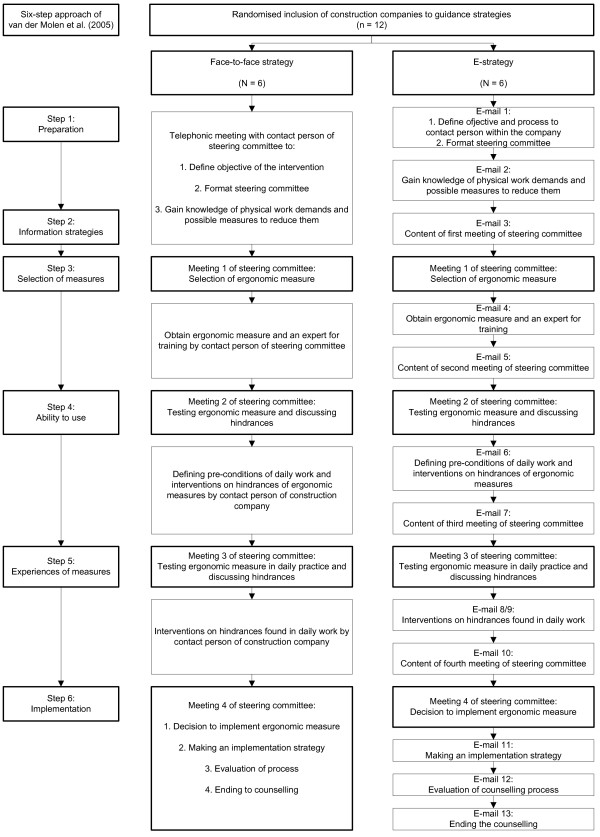
**Overview six-step approach.** Figure 1: An overview of the six-step approach of the PE intervention of van der Molen *et al*. [12]. In addition, an overview of the two guidance strategies for the PE intervention is shown. All emails will be send to the contact person of the steering committee.

The intervention is based on the PE intervention used by van der Molen *et al*. [12] but has been adapted with respect to the guidance strategies. The original PE intervention consisted of a six-step approach in which different company stakeholders (i.e., the CEO, the prevention worker, work planners, foremen, and construction workers (i.e., blue-collar workers)) participated. In the intervention of this study, two ergonomics consultants make, based on the original six-step approach, two protocols of the PE intervention, one for the face-to-face guidance strategy, in which the intervention is guided through one telephone contact and four personal contacts of the ergonomics consultant, and one for the e-guidance strategy, in which the intervention is guided with the help of 13 email contacts. An overview of both guidance strategies is given in Figure 1. During the intervention, costs concerning the guidance by the ergonomics consultants is paid out of research budget. Construction companies are free to choose which ergonomic measure they wish to implement. The maximum duration of each guidance strategy is six months.

#### Face-to-face guidance strategy

The face-to-face guidance strategy consists of guiding the steering committee through the six-step approach during four meetings within six months. Before the first meeting, the steering committee has to be established and financial budget for the intervention has to be reserved. Establishing the steering committee will be done by the contact person of the steering group, the CEO or the prevention worker, within the company after a telephone meeting with the ergonomics consultant, this being step 1a of the six-step approach. Members of the steering committee will be the CEO, the prevention worker, work planners, foremen, and construction workers, and although the members of the steering committee are defined as different stakeholders, one person may fulfil different stakeholder roles. During this telephone meeting, a first meeting of the steering committee, in which the ergonomics consultant takes part, will be planned.

Within the same telephone meeting and in advance of the first meeting, the contact person will have to collect information on physical work demands of the chosen occupation and possible ergonomic measures to reduce the physical work demands (step 2). This can be done through research on the internet, code of practice or by consulting the construction workers of the chosen occupation. An overview of the physical work demands will be made by the contact person, describing what the physical work demand at risk is, the category of the physical work demand, i.e. horizontal transportation, vertical transportation, or positioning of materials [20], and the possibility to reduce the physical work demands.

The prime purpose of the first meeting is to discuss the objective and the process of the intervention – step 1b of the six-step approach. At the end of the meeting, an ergonomic measure will be selected to be implemented through tailored information on measures and discussing the advantages and disadvantages of the ergonomic measures (step 3). The choice of an ergonomic measure will be facilitated by a self-made scorecard listing the measure, (e.g. the availability of the measure), the behaviour of the construction workers (e.g. need for training to work with the measure), and the organisation (e.g. the possibility to purchase the measure at short notice). Each level contains three statements.

Prior to the second meeting, the contact person must arrange a meeting with the supplier/manufacturer of the selected ergonomic measure. Furthermore, a test environment must be arranged to facilitate the ability to acquire experience working with the chosen ergonomic measure. During the second meeting, construction workers will be able to get instruction and training on the ergonomic measure by an expert in a test environment (step 4). The expert may be a worker within the company who is familiar with the ergonomic measure, or the supplier of the ergonomic measure. Interventions regarding any hindrances on the use of the ergonomic measures will be discussed. In addition, instrumental help through a self-performed cost-benefit analysis of the ergonomic measure by the ergonomics consultant will be done to gain insight into the financial consequences for the companies.

The third meeting is intended to test the ergonomic measure in daily practice (step 5). Prior to this meeting, the interventions on the hindrances must have been carried out and preconditions for the test location in practice must have been formulated. During the third meeting, experiences of the ergonomic measures in daily work will be discussed. This results in interventions on hindrances in, and adaptations of, the preconditions of daily practice.

The interventions on hindrances in, and adaptations of, the preconditions will form the input for the final meeting and the last step (step 6). In this meeting, the test in practice will be evaluated and a choice will be made regarding whether the ergonomic measure will be implemented. An implementation strategy will be drawn up for the construction company, based on three levels: measure, e.g. buying or hiring the measures; behaviour, e.g. training of all construction workers; and organisation, e.g. deciding in which projects the ergonomic measure will be used. In addition, all four meetings will be evaluated, and members of the steering committee will be asked whether all four meetings are useful.

Once all the meetings have been completed, all of the construction workers within the chosen occupations will be informed on the process of the intervention. In this manner, construction workers who are not part of the steering committee will be able to give input throughout the entire intervention. With the exception of the preparation of the first meeting, all assignments in the preparation of a steering committee meeting will be given through personal contact of the ergonomics consultant with the contact person of the steering committee at the end of the last meeting.

#### E-guidance strategy

The e-guidance strategy consists of guiding the steering committee through the six-step approach in 13 emails. The content of the first email contact is to make clear the objective and process of the intervention to the contact person, the CEO or prevention worker, within the company. A document describing the entire protocol is therefore attached to the first mail. In addition, the contact person will be assigned to establish a steering committee with the different company stakeholders (i.e. the CEO, prevention worker, work planners, foremen, and construction workers (i.e., blue-collar workers)), and to reserve financial budget for the intervention (step 1a).

The objective of the second email to the contact person is to collect information on physical work demands of the chosen occupation and on ergonomic measures to reduce the physical work demands in preparation for the first meeting of the steering committee (step 2). This can be done through research on the internet, a study of the code of practice or by consulting the construction workers. An overview of the physical work demands will be sent back by the contact person to the ergonomics consultant, describing what the physical work demands at risk are, the category of the physical work demand, i.e. horizontal transportation; vertical transportation; or positioning of materials [20], and the possibility to reduce the physical work demands.

The third email contains the agenda of the first meeting of the steering committee. The ergonomics consultant will not be present during this meeting. The main objective will be discussed, as well as the process of the intervention (step 1b). An ergonomic measure will be selected at the end of the meeting through tailored information on measures and discussing the advantages and disadvantages of the ergonomic measures (step 3). The selection will be facilitated by a self-made scorecard with respect to the measure (e.g. availability of the measure), the behaviour of the construction workers (e.g. need for training to work with the measure), and the organisation (e.g. possibility to purchase the measure at short notice). Each level contains three statements.

The preparation of the second meeting is the content of the fourth email. Within the second meeting, the selected ergonomic measure will be tested by the construction workers in a test environment without any financial risks. The preparation for this meeting consists of making an agreement with the supplier/manufacturer of the selected ergonomic measure to acquire the selected ergonomic measure and to arrange an expert of the ergonomic measure to instruct and train the construction workers. In addition to the supplier/manufacturer, the expert can be a worker within the company who is familiar with the ergonomic measure, or the supplier or manufacturer of the ergonomic measure.

The fifth email provides the agenda of the second meeting. During the second meeting, construction workers will be able to get instruction and training on the ergonomic measure by the expert in a test environment (step 4). Hindrances to the use of ergonomic measures will be discussed and interventions to overcome these hindrances will be discussed by the steering committee. In addition, instrumental assistance through a self-performed cost-benefit analysis of the ergonomic measure by the ergonomics consultant will be performed to get insight into the financial consequences for the companies.

The preparation for the third meeting will be the content of the sixth email. Preconditions for the test location in practice will be defined by the contact person and interventions on the hindrances, as a result of the second meeting, will be carried out. In addition, a test location will be selected for the third meeting.

During the seventh email contact, the objective of the third meeting will be set. The third meeting is to test the ergonomic measure in daily work (step 5). Experiences of advantages and disadvantages on the part of the construction workers on the ergonomic measures in daily work will be identified by the steering committee. Emails eight and nine concern the interventions on hindrances and adaptations to the preconditions of daily work.

The tenth email contact provides the agenda for the fourth meeting. Based on the test in daily work, a discussion will take place regarding whether the ergonomic measure will be implemented. In addition, points of interest will be defined for the further implementation. During the 11th email contact, an implementation strategy will be drawn up for the construction company based on three levels: measure, e.g. buying of hiring the measures; behaviour e.g. training of all construction workers; and organisation, e.g. deciding in which projects the ergonomic measure will be used (step 6).

After drawing up the implementation strategy, the process will be evaluated in email contact 12. Of each e-mail contact, the contact person will be asked whether the protocol is useful for the implementation of ergonomic measures. The 13th and final email contact contains the ending of the PE process.

After each meeting of the steering committee, all construction workers of the chosen occupations will be informed about the process of the intervention. In this manner, construction workers who are not part of the steering committee will be able to provide input throughout the entire intervention. The information obtained from each assignment given in each email contact will be returned by email to the ergonomics consultant. In each subsequent email contact of the ergonomics consultant, feedback will be given on the obtained information by the ergonomics consultant to the contact of the steering committee.

#### Co-interventions

No co-interventions are planned by the participating construction companies during this intervention which might influence the use of new ergonomic measures by their employees.

### Outcome measures

The outcome measures will be measured by questionnaires at baseline and six-months after baseline. Baseline is defined as the start of the intervention, just before the first contact between the ergonomics consultant and the research coordinator in the company.

#### Primary

The use of ergonomic measures is the primary outcome measure and is defined as whether ergonomic measures are used during the last two months by the individual construction workers. The ergonomic measures are defined in four clusters: 1) mechanical measures for horizontal or vertical transportation, such as a crane, 2) ergonomic measures for raising equipment or materials, such as a raised mortar hod, 3) ergonomic measures to adjust working height on the worksite, and 4) ergonomic handtools. Construction workers will be asked at baseline and after six months whether they used ergonomic measures during the last two months during their work (yes or no). Examples of the ergonomic measures are adjusted for the different occupations.

In addition, construction workers are asked at the end of the intervention how many days they could and did use the implemented ergonomic measure during the previous 10 working days.

#### Secondary

##### Work ability

The measurement of work ability will be determined at baseline and after six months with the first three items of the Work Ability Index (wai)[21]: 1) current work ability compared with the lifetime best; 2) perceived work ability with respect to physical demands; and 3) perceived work ability with respect to mental demands.

##### Physical functioning

Physical functioning is assessed at baseline and after six months, and defined as limitations during 10 activities during a typical day, for instance climbing several flights of stairs or walking several hundred yards. Individual construction workers will be asked to state how much their health currently limits them in these 10 activities. The measurement will be determined with the subscale ‘physical functioning’ of the Dutch translation of the mos-36 questionnaire, the rand-36[22]. The categories for the answers are for each activity: ‘yes, serious limited’, ‘partly limited’, and ‘not limited at all’.

##### Limitations due to physical problems

Limitations due to physical problems are defined as whether individual construction workers had 1) spent less time on their work, 2) achieved less in their work, 3) were limited in and 4) had difficulty with their work or other regular daily activities as a result of their physical health. These four items will be measured with the subscale ‘limitations due to physical problems’ of the rand-36[22]. Each of the four statements must be answered with ‘yes’ or ‘no’.

#### Descriptive variables

The demographic characteristics, job characteristics and education will be assessed at baseline. The demographic characteristics are gender, and age (in years). The job characteristics are work experience in years as a construction worker, work experience in years in the current occupation, and whether the construction worker is in a managerial position. For education, the highest completed education level will be asked, divided into three categories: primary education, secondary (vocational) education or higher professional education. All descriptive variables will be assessed with self-formulated questions.

#### Process evaluation

The process evaluation of the two guidance strategies will be determined using indicators as defined by Linnan and Steckler [23] and Murta *et al*. [24]. The following process- and performance indicators will be evaluated: reach, dose delivered, dose received, precision, competence and satisfaction. An additional process indicator is the behavioural change of the construction workers.

Reach is defined as the attendance rate of the construction companies at the intervention. Attendance is defined as the number of construction companies participating in this study relative to the number of construction companies invited through the recruitment strategies. The attendance will be assessed by means of a logbook during the recruitment of the construction companies. Construction companies that will be contacted by the researcher (SV) and do not want to participate will be asked to explain why. Dose delivered refers to the amount or proportion of the intended intervention that is actually delivered to the participating contact persons of the construction companies. Within the six steps of the PE intervention, the dose delivered will be assessed by 31 performance indicators defined by van der Molen *et al*. [12]. Of these 31 performance indicators, 19 are defined as essential. The number of all performance indicators and of the 19 essential performance indicators delivered by the assignments of the ergonomics consultants to the steering committees will be assessed during the face-to-face guidance strategy by means of a logbook filled by an observer who is present at the meetings of the steering committee. For the e-guidance strategy, dose delivered will be assessed by means of the content of the emails from the ergonomics consultant to the contact person. Dose delivered is considered sufficient when at least all 19 essential performance indicators are delivered. When companies drop out of the study or do not follow the entire intervention, reasons why will be asked by the researcher (SV).

In addition, six performance indicators are defined for dose delivered of the steering committee to all construction workers within the included companies. The performance indicators are: 1) received information from the steering committee on the objective of the project, 2) received information on musculoskeletal complaints within the occupation, 3) received information on ergonomic measures, 4) involved with the choice of an ergonomic measure, 5) received information and training of the chosen ergonomic measure, and 6) tested the ergonomic measure in daily work. Dose delivered to the construction workers will be assessed by acquiring all information sent from the steering committee to the construction workers and via attendance lists during the meetings of the steering committee.

Dose received refers to the proportion of activities in the intervention that is actually performed by the steering committees of the construction companies. The performance indicators will be assessed by an observer by means of a logbook during the meetings of the steering committee during the face-to-face guidance strategy. For the e-guidance strategy, dose received will be assessed by means of a logbook by an observer who is present at the meetings of the steering committee and by means of the feedback of the assignments of the steering committees to the ergonomics consultant. Dose received is defined as sufficient when at least the 19 essential performance indicators are performed by the steering committee. Whether the dose is received by construction workers was assessed by seven questions about the seven performance indicators within the questionnaire after six months. In addition to the six performance indicators described above, an extra performance indicator is added to assess whether construction workers had read the delivered information. Dose received is sufficient when all seven performance indicators are received.

The precision of the intervention is defined as whether construction companies implement ergonomic measures described by the websites of Arbouw or codes of practice. If the implemented ergonomic measures are described by these websites or catalogues, the required precision is considered to have been reached.

The question of whether the ergonomics consultant has the competence to guide the steering committees of the construction companies will be asked with the help of a questionnaire after the six months of guidance has been completed. The contact person will be asked whether the assignments in preparation of the meetings were clear, whether the objectives of the four meetings were clear, whether the objectives of the feedback of the assignments to the ergonomics consultant were clear, whether the questions asked by the contact person were answered satisfactorily, and whether the ergonomics consultant was able to help with problems occurring during the six months of guidance. All items will be answered with yes or no and additional information on the given answer will be requested.

After six months, the company stakeholders within the steering committee are asked via a questionnaire whether they were satisfied with the guidance strategy and if it had been of value for the construction company. The questionnaire contains seven items, including the duration of the intervention, the duration of the meetings, the involvement of construction workers with the choice of an ergonomic measure. In addition, with two open-ended questions, members of the steering committee can give suggestions for improvements to the intervention, to the guidance strategy, and to the consultant. With the exception of the duration of the intervention and the open-ended questions, all items will be answered with yes or no. For all questions, additional information on the given answer will be requested.

The behavioural change of the construction workers consists of six topics: knowledge, attitude, motivation, ability to use, facilitation and culture. It is considered that the interventions will change the behaviour of construction workers towards working with ergonomic measures. Therefore, measurements of the items for behavioural change will be done at baseline and after six months by means of a self-made questionnaire.

First of all, the knowledge of the relationship between ergonomic measures, physical work demands and musculoskeletal disorders will be asked through two statements. An example of a statement is: “Due to long and frequent lifting and bending, I have an increased risk of low back pain. This can be reduced by using a raised hod”. The statements will be adapted for the different occupations, and construction workers will be asked if they agree with the statement. They can answer with a ‘yes’ , ‘no’ or ‘I don’t know’. Knowledge will be rated as good when both questions are answered affirmatively, and knowledge within a construction company is defined as good when the knowledge of 75% of all the construction workers within the company is rated as good.

The attitude of the construction workers towards working with ergonomic measures is asked with five yes or no items, such as “I only use ergonomic measures when I have physical complaints”, and is defined as good when four of the five items are scored positively. On the company level, attitude is considered good when at least 75% of the construction workers scored positively.

The motivation to work with ergonomic measures is asked through a single yes or no question. This question is: “When your employer provides ergonomic measures, are you willing to use them?” If the question is answered in the affirmative, construction workers are considered to have the motivation to work with ergonomic measures. Motivation is considered as good at company level when at least 75% of the construction workers answered the question in the affirmative.

Of the four earlier defined clusters of ergonomic measures, the ability to use these ergonomic measures will be asked. For each cluster, construction workers can assess their ability as good or poor.

As with the ability to use, the facilitation of each of four clusters of ergonomic measures will be assessed. In addition, a question is asked concerning whether the construction company has set up rules or procedures for the use of ergonomic measures.

The final topic of behavioural change is the culture of the construction company. By means of three items, the values and expectations of construction companies towards working with ergonomic measures will be asked. An example of such an item is: “It is expected of me and my colleagues that we work with ergonomic measures as much as possible”. The culture of the construction company is positive for an individual construction worker when all three items are answered affirmatively. At least 75% of the construction workers must experience a positive culture for the construction company to be considered as possessing a positive culture.

#### Economic evaluation

A cost-benefit analysis for each participating construction company will be made. At baseline, demographic characteristics of the employees with respect to sick leave will be assessed for the construction companies. The costs of the intervention will be assessed at the end of the intervention by multiplying the time spent by the ergonomics consultant with the hourly costs. In addition, at the end of the intervention, the director of the construction companies will be asked about the costs regarding employing the ergonomic measures and additional energy and the maintenance costs of the ergonomic measures. Furthermore, the director is asked if the production per working hour changed by using the ergonomic measure.

On the benefit side, construction workers will be asked at the end of the intervention how many days they could and did use the ergonomic measure during the previous 10 working days. In addition, construction workers are asked how they judge their physical work demands compared to working without the ergonomic measure. These three questions will provide a benefit for reducing risk factors for work-related musculoskeletal disorders. Another benefit aspect will be the change in production per working hour and change in quality of the work.

With the help of the Dutch Economic Institute of the Construction Sector, the above-mentioned aspects on the company level and on the individual worker level will result in a cost-benefit analysis for each construction company.

### Sample size

The sample size is based on detecting a difference in the use of a new ergonomic measure between the face-to-face guidance strategy and the e-guidance strategy. In a study of Jensen and Friche [6], it was found that daily use of new introduced construction working methods could be at least 10%. We have decided that a 15% absolute difference in use of newly introduced ergonomic measures between the two guidance strategies is a realistic and relevant difference.

Based on a power calculation using the Nquery Advisor software [25], 113 workers per group must be included for a difference of 15% (from 10% to 25%) with an alpha of 0.05 (two-tailed) and a power of (1-beta) = 0.80. A small cluster effect on the use of ergonomic measures at the level of the construction companies will be anticipated (ICC = 0.01). The construction companies have influence on the use of ergonomic measure by their employees, therefore there is a correlation between company and its employees. Since there is no interaction between the participating companies and because of the different characteristics of the companies (e.g. culture within the company, different trades) the assumption is that the correlation between the companies will be small. Based on the inclusion criteria, the assumption is made that the average number of construction workers (n) within a construction company is on average 25. This results in a cluster effect of 1.24 (1 + ((n-1) × ICC) and an adjusted sample size of 140 per group. To reach this sample size, we must include six construction companies per group.

### Randomisation

The construction companies will be block randomised with a block size of two with the help of nQuery Advisor 7, a computer generated randomisation table. The independent variable for this randomisation is the sequence of inclusion. The first construction company participating within an occupation will be randomly assigned to the face-to-face guidance strategy group or the e-guidance strategy group. The second company participating within the same occupation will be assigned to the opposite strategy group. The implementation strategy makes blind group assignment impossible for the participating companies, the ergonomics consultants and the observers of the steering committee meetings.

Based on practical constraints, the ergonomics consultants divided the Netherlands into a northern part and a southern part. Construction companies in the northern part of the Netherlands who are assigned into the face-to-face guidance strategy group are allocated to ergonomics consultant 1, and construction companies in the southern part to ergonomics consultant 2. Construction companies assigned into the e-guidance strategy group are randomly assigned to ergonomics consultant 1 or 2.

### Statistical analyses

#### Effect evaluation

Descriptive variables, primary and secondary outcome measures at baseline and after six months will be presented for the two guidance strategies per construction company using descriptive statistics. Differences in descriptive variables between the two guidance strategies will be tested using an independent samples *t*-test for continuous data or a Chi-square test for proportions.

The results of the primary and secondary outcome measures on the individual worker level may be influenced by the dependency of the company in which an individual worker is working. To correct for this dependency, the statistical analyses of the outcome measures to test differences between baseline and after six months for both guidance strategies will be done using a Generalized Linear Mixed Model. IBM SPSS Statistics 20 will be used for the statistical tests.

#### Process evaluation

With the exception of the behavioural change, each topic of the process evaluation will be reported descriptively on the company level and on the individual level. The items of the behavioural change will be pre-post tested using a General Linear Mixed Model. IBM SPSS Statistics 20 will be used for the statistical tests.

#### Economic evaluation

The costs-benefit calculation for each company will be analysed descriptively on the company level. The financial costs and benefits will be given for each company.

## Discussion

The results of this study will help to gain insight into the effectiveness and possible barriers and facilitators of the two guidance strategies of a PE intervention on the use of ergonomic measures, work ability, physical functioning, limitations due to physical problems, process evaluation and economic evaluation. If shown to be effective in terms of an increase in the use of ergonomic measures, one or both strategies can be implemented by occupational health services to help construction companies or other companies with the implementation of ergonomic measures by means of an appropriate guidance strategy.

### Methodological considerations

#### Strengths

For both guidance strategies, a protocol was developed based on the six-step approach of van der Molen *et al*. [12]. The presence of such a protocol was found in 17 out of 18 studies as a facilitator for the PE intervention in the review of van Eerd *et al*. [16]. Other possible barriers to and facilitators of the PE intervention that are found in more studies within the review of van Eerd *et al*. [16] and by Driessen *et al*. [26] at organisational level are within the protocol of the guidance strategies.

In addition, construction companies are free to choose which ergonomic measure they want to implement. By selecting an ergonomic measure in the early stage of the protocol, and with the help of the self-made scorecard of the ergonomics consultants, construction companies have an overview of which actions are important for facilitating the implementation process of the ergonomic measure.

Since earlier studies on the effectiveness of PE interventions on the use of ergonomic measures found contradictory results, we have added health outcomes as secondary outcome measures. Throughout the duration of this study, we held the belief that the presence of musculoskeletal disorders among construction workers would not change. This is why we look at more proxy outcome measures for health outcomes. The ergonomic measures reduce physical work demands, and we believe that this is reflected in the short term on work ability, physical functioning and limitations due to physical problems.

#### Limitations

One could argue that a lack of a control group could be seen as a limitation, since this is, in the context of the evidence-based health care, considered as the gold standard for providing evidence for the effectiveness of an intervention [19]. However, in this study we are interested in the effectiveness of the guidance strategies rather than of the PE intervention, since the effectiveness of a PE intervention has been the subject of many studies [11,14-16]. The next step is to assess information on the best practice of the guidance of a PE intervention.

The wide diversity of the occupations within this study will make it easier to generalise the outcomes of this study towards other occupations or other sectors. However, it might also influence the outcome of this intervention. The construction industry is a diverse sector and a continuous process in which many occupations must work at the same time. Therefore, low-cost individually-oriented measures, such as handtools, are probably easier to implement than costly technical equipment with organisational consequences on worksites. We tried to remove this hindrance through block randomisation of the construction companies within the same occupation. However, this only works when an even number of construction companies within the same occupation are included.

An obstacle for the recruitment of construction companies might be the economic recession. In 2012, many construction companies went bankrupt. Besides influencing the recruitment of the construction companies, this might also influence the outcome since the priority of the construction companies will not lie with the implementation of ergonomic measures. By setting the duration of the guidance at six months, we provide the construction companies sufficient time to follow the PE process and maintain enough time for their core business.

The results of the present study will be available in 2014.

## Abbreviations

PE: Participatory ergonomics.

## Competing interests

The authors declare that they have no competing interests.

## Authors’ contributions

SV is responsible for data collection and drafted the manuscript. All authors conceived and designed the study, read and corrected draft versions of the manuscript and approved the final manuscript. HM, JS and MFD obtained funding for this study.

## Pre-publication history

The pre-publication history for this paper can be accessed here:

http://www.biomedcentral.com/1471-2474/15/132/prepub
